# Evaluation of a telehealth service to support breast cancer prevention medication uptake: a protocol of a mixed methods study

**DOI:** 10.1136/bmjopen-2024-098198

**Published:** 2025-06-18

**Authors:** Katrina Louise West, Tim Spelman, Wanda Cui, Sarah Latham, Sabine Deij, Sandy Minck, Sarah Mason, Christobel Saunders, Catherine Poliness, Paul A James, Greg Wheeler, Louise A Keogh, Stephanie Best, Kelly Anne Phillips

**Affiliations:** 1 Department of Medical Oncology, Peter MacCallum Cancer Centre, Melbourne, Victoria, Australia; 2 The Sir Peter MacCallum Department of Oncology, The University of Melbourne, Melbourne, Victoria, Australia; 3 Centre for Health Services Research and Implementation Science, Peter MacCallum Cancer Centre, Melbourne, Victoria, Australia; 4 Burnet Institute, Melbourne, Victoria, Australia; 5 Clinical Informatics, WEHI, Melbourne, Victoria, Australia; 6 Department of Surgery, The University of Melbourne Melbourne Medical School, Melbourne, Victoria, Australia; 7 Centre for Health Policy, The University of Melbourne School of Population and Global Health, Melbourne, Victoria, Australia; 8 Division of Research, Peter MacCallum Cancer Centre, Melbourne, Victoria, Australia; 9 Department of General Surgery, The Royal Melbourne Hospital, Melbourne, Victoria, Australia; 10 Department of Cancer Surgery, Peter MacCallum Cancer Centre, Melbourne, Victoria, Australia; 11 Parkville Familial Cancer Centre, Peter MacCallum Cancer Centre and the Royal Melbourne Hospital, Parkville, Victoria, Australia; 12 Department of Radiation Oncology, Peter MacCallum Cancer Centre, Melbourne, Victoria, Australia; 13 Centre for Health Equity, The University of Melbourne School of Population and Global Health, Melbourne, Victoria, Australia; 14 Melbourne School of Health Sciences, The University of Melbourne, Melbourne, Victoria, Australia; 15 Australian Genomic, Murdoch Children’s Research Institute, Parkville, Victoria, Australia; 16 Centre for Epidemiology and Biostatistics, The University of Melbourne School of Population and Global Health, Melbourne, Victoria, Australia

**Keywords:** Breast tumours, Telemedicine, Primary Prevention, Preventive Health Services

## Abstract

**Introduction:**

Breast cancer risk can be substantially reduced with risk-reducing medications (RRMeds). Despite their efficacy, and guidelines which support their use for women at substantially increased risk of breast cancer, they are underused. Barriers to their use in Australia include a lack of awareness of RRMeds by women and clinicians, and a primary care workforce that reports a lack of knowledge and confidence in discussing and/or prescribing these medications. In contrast, Australian clinicians have reported specialist support and guidance as a key facilitator. The Preventing Cancer with Medications (PCMed) Telehealth Service was therefore developed to provide this specialist support and to bridge the evidence–implementation gap. The PCMed Service endeavours to increase the appropriate use of RRMeds and support women and their doctors throughout treatment. The aim of this research is to evaluate the effectiveness, adoption, acceptability, feasibility, fidelity and cost of this new Service, and to determine any adaptations that might be required.

**Methods and analysis:**

The research uses a mixed methods approach. Effectiveness of the PCMed Service will be evaluated by determining whether the PCMed Service is associated with increased uptake of RRMeds compared with historical data. Secondary outcomes include: adoption of the Service, specifically, the proportion of women who attend a PCMed Service consultation; acceptability of the Service for clients and referring clinicians (using a brief survey and semistructured interviews); feasibility and fidelity by evaluating the adherence to the planned Service processes; and the cost, by reporting the difference between funding received per woman and the cost for service delivery.

**Ethics and dissemination:**

This study was approved by the institutional Human Research Ethics Committee (EC00235): HREC/101142/PMCC. The findings will inform future iterations of the Service prior to scaling up. Research findings will be disseminated at scientific meetings and in peer-reviewed journals.

**Trial registration number:**

ISRCTN15718519.

**STRENGTHS AND LIMITATIONS OF THIS STUDY**

- A key strength is the theory-informed implementation data collected from participants (clients and clinicians) and the inclusion of cost analyses to aid the future development of the Preventing Cancer with Medications (PCMed) Service.
- The inclusion of a historical cohort from the same clinics as the prospective cohort (for the primary aim), rather than relying on published data for the preimplementation comparator rates of RRMed uptake is also an important strength.
- Implementation of the PCMed Service and evaluation are taking place in one large Australian Cancer Centre, thus potentially limiting the generalisability of the findings.

## Introduction

 Globally, breast cancer is the most common cancer in women. In 2020, 2.3 million women were diagnosed with breast cancer and 685 000 women died of the disease.^1^ If the current global trends continue, it is expected that there will be 3 million new cases of breast cancer occurring each year by 2040.^2^ Importantly, this upward trend is not inevitable, as many of these breast cancers are preventable.^3^

Optimal implementation of proven, intensified prevention strategies for women at substantially increased risk of breast cancer, combined with minimising women’s exposure to modifiable risk factors (eg, obesity, alcohol and sedentary lifestyle), will be crucial to lower breast cancer incidence.^3 4^ Approximately 50% of breast cancers occur in the 20% of women who are at increased risk.^5^

**Figure 1:**
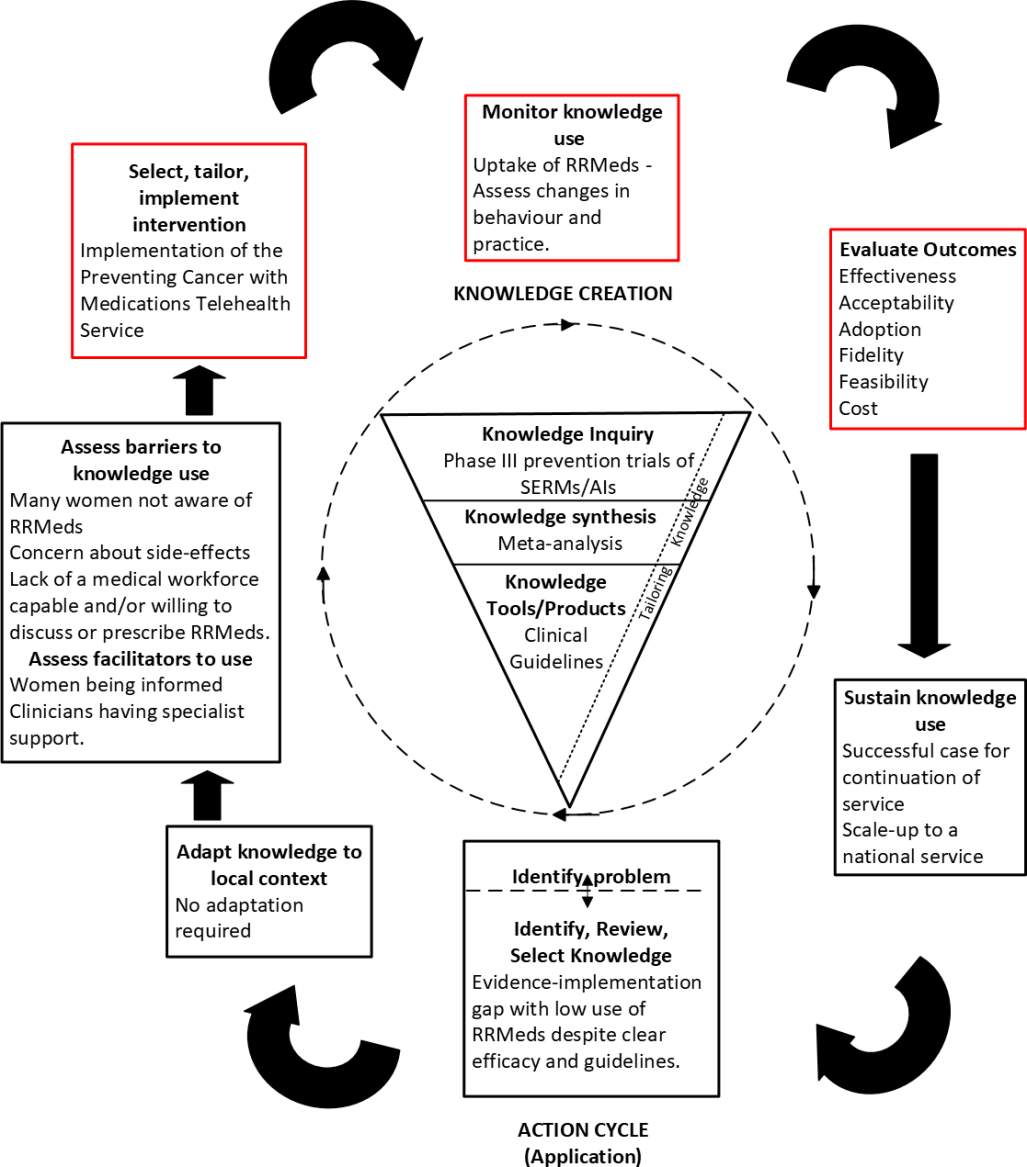
Knowledge creation, as it pertains to the benefits and risks of RRMeds, supports the use of RRMeds as a standard of care. Barriers and facilitators to the uptake of RRMeds have been identified. This research focuses on the steps highlighted in red. AIs, aromatase inhibitors; RRMeds, risk-reducing medications; SERMs, selective oestrogen receptor modulators.

Several medications reduce the risk of oestrogen receptor-positive breast cancer.^6^,^17^ These medications include the selective oestrogen receptor modulators (SERMs), tamoxifen and raloxifene, and the aromatase inhibitors (AIs), anastrozole and exemestane (all hereafter referred to as risk-reducing medications (RRMeds)). Guidelines recommend their consideration in individuals who are at moderate to high risk of developing a future breast cancer,^18^,^23^ excluding those with contraindications such as pregnancy, planning pregnancy in the next 3 years, breastfeeding, prior bilateral mastectomy or use of hormone therapy. Additional contraindications include prior thromboembolic events, endometrial cancer (for tamoxifen), smoking, taking anticoagulants or a strong inhibitor of the CYP2D6 enzyme (for SERMs) and premenopausal status or osteoporosis (for AIs).^24^ A single tablet, taken daily for 3–5 years, can reduce the relative risk of breast cancer by between 30% and 60%,^6^,^17^ and the risk reduction persists long after the prescribed treatment period.^8 13 14 16^ Despite strong evidence of efficacy and endorsement in national guidelines,^21^,^23^ the use of RRMeds in Australia is low.^25^ A major barrier to the use of RRMeds is the concern for side effects.^26 27^ Common adverse effects include gynaecological and vasomotor symptoms, menstrual changes, and less commonly, more serious events such as thromboembolism (with SERMs) or endometrial cancer in postmenopausal women (with tamoxifen),^6^ or bone loss (with AIs).^10^ Confusion between RRMeds and chemotherapy,^26 27^ and insufficient information for women to make an informed decision regarding their use,^26^,^30^ suggest that expert medical advice is important for women to weigh the potential benefits and harms in the context of individual risk. Many Australian women at increased risk of breast cancer, and approximately one-third of their primary care physicians (PCPs), are unaware of RRMeds.^27^ Another driver of this evidence–implementation gap in Australia is the lack of a medical workforce capable of, and willing to discuss and prescribe RRMeds.^27^ In one study, among PCPs who were aware of RRMeds, only 3% were very confident in discussing them, and 31% considered it their role to initiate prescribing, although 98% of PCPs would write ongoing prescriptions.^27^ Specialist support has been identified as a key facilitator for PCPs to discuss and prescribe RRMeds.^27^ In response to this, the Preventing Cancer with Medications (PCMed) Telehealth Service has been developed to provide specialist support to PCPs. The ‘knowledge to action’ framework^31^ (figure 1) was adapted to guide the development and implementation of the PCMed Service and its concurrent evaluation.

We hypothesise that the implementation of the PCMed Service will be associated with greater use of RRMeds compared with historical controls, and the PCMed Service will be feasible, well-adopted, delivered with fidelity and acceptable to both clients who attend the PCMed Service and to clinicians.

### Intervention—novel PCMed Service

**Figure 2:**
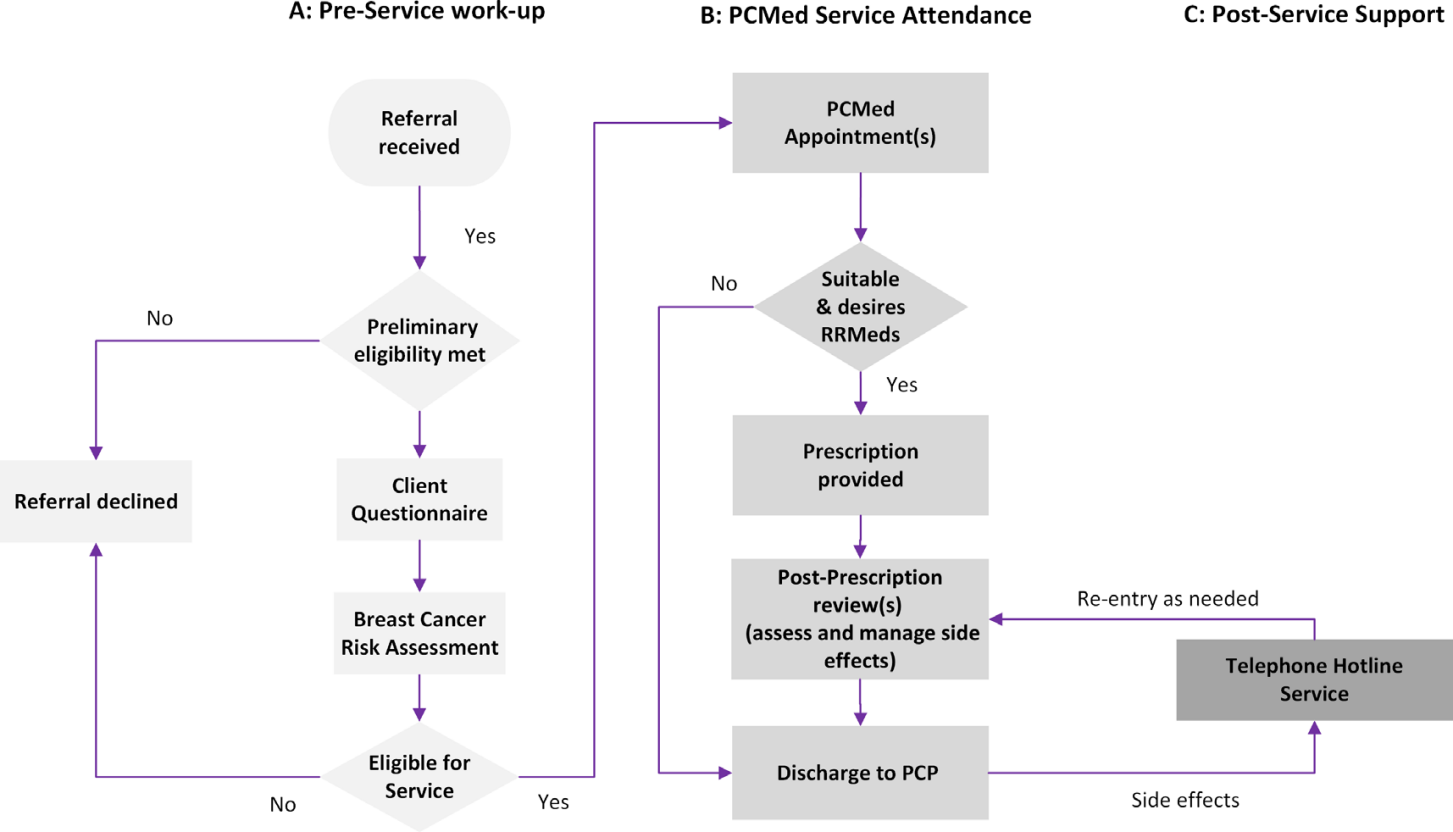
(A) Preservice work-up: referrals are reviewed by the nurse practitioner (KLW). Women who meet preliminary eligibility criteria are sent a questionnaire (regarding demographics, social and medical history, breast cancer risk factors and willingness to consider RRMeds). Breast cancer risk is determined using validated risk prediction models/tools such as iPrevent,^32^ Tyrer Cuzick,^33 34^ or the Breast and Ovarian Analysis of Disease Incidence and Carrier Estimation Algorithm (BOADICEA)^35 36^,unless there is a history of thoracic radiotherapy, in which case risk is estimated using published data.^37^ For women referred from services external to catchment clinics, the iPrevent^32^ prediction model is applied. Eligible women are offered an appointment in the Service. (B) PCMed Service attendance: in the initial telehealth appointment, clients receive a discussion of their personalised breast cancer risk. If a client desires, their 10-year, 20-year or remaining lifetime risk is conveyed as a numerical value. The names and mechanism of action of potentially appropriate RRMeds, the absolute risk reduction that could be achieved with RRMeds, and other benefits and any potential adverse effects of RRMeds are discussed. Client understanding of the information received is assessed. A second consultation is offered for clients requiring more time to decide on whether to take RRMeds. Clients who desire RRMeds receive a prescription and are booked for review in 8–10 weeks to assess and manage any side effects. If tolerating the RRMed, care is transferred to the PCP with additional information and instructions to continue management. (C) Postservice support: a hotline telephone service is provided for clinicians and clients to address any concerns relating to side effects, and additional appointments are made if required. PCMed, Preventing Cancer with Medications; PCP, primary care physician; RRMeds, risk-reducing medications.

The PCMed Telehealth Service at the Peter MacCallum Cancer Centre (PMCC) in Melbourne is the first of its kind in Australia. It is co-led by a medical oncologist (KAP) and a nurse practitioner (KLW). It is the only specialist service in Australia solely dedicated to delivering comprehensive, personalised information to women to facilitate their informed decision-making about using RRMeds. For women who are suitable and choose to take RRMeds, an initial prescription is provided. The woman, her referring clinician and PCP are offered access to ongoing support to promote sustained use of RRMeds. The PCMed Service intervention is detailed in figure 2.

Delivered by telehealth, the PCMed Service is intended for women at substantially increased risk of a first breast cancer who are interested in considering taking medications to reduce their risk. Referrals are accepted from clinicians from the PMCC and surrounding services who routinely see women at increased risk of breast cancer. These referring clinics will henceforth be referred to as ‘catchment clinics’; they include Late Effects, Breast Surgical and Familial Cancer Clinics at PMCC and the Familial Cancer Clinic at the Royal Melbourne Hospital. Referrals are also accepted from clinics external to the catchment clinics and from PCPs. Women who attend the PCMed Service are referred to as clients.

### Aims

The overarching aim is to evaluate the effectiveness and process of implementing this new clinical service.

The primary aim is:

- To determine whether the PCMed Service is associated with an increased use of RRMeds by women who attend catchment clinics, compared with use by similar women who attended catchment clinics before the implementation of the PCMed Service.

Secondary aims include:

- To describe the adoption of the PCMed Service by women who attend catchment clinics. Specifically, the proportion of eligible women who attend the PCMed Service following a catchment clinic appointment where the PCMed Service is discussed.
- To determine the uptake of RRMeds for all clients who attend the PCMed Service and the associated characteristics.
- To explore the reasons for not taking up RRMeds and the associated characteristics.
- To explore the acceptability of the PCMed Service to clients.
- To explore the acceptability of the PCMed Service to clinicians.
- To determine the feasibility and fidelity of the PCMed Service intervention and any adaptations required.
- To evaluate the cost of delivering the PCMed Service from the perspective of the healthcare provider.

## Methods and analysis

### Study design

The process evaluation described here is evaluating this new complex intervention (the PCMed Service) using a mixed methods (qualitative and quantitative) approach. The study has prospective and retrospective components.

### Recruitment to the PCMed Service and study

Prior to the commencement of the PCMed Service, clinicians working in the catchment clinics, including breast care nurses, were informed of the PCMed Service and its concurrent evaluation. Resources were developed and placed in the catchment clinics. These include a client brochure and a clinician fact sheet that describes the Service, eligibility criteria and the referral process.

Eligible women are actively recruited for the PCMed Service from catchment clinics by the nurse practitioner (KLW), who reviews the electronic medical records of women in advance of their catchment clinic appointment and identifies those who are potentially eligible for the PCMed Service. Clinicians seeing these women are reminded by KLW of the PCMed Service and asked to provide eligible women with a client brochure and to refer them to the PCMed Service if appropriate.

#### Inclusion criteria for the study population

Women are eligible if they have no personal history of invasive breast cancer or ductal carcinoma in situ, are aged between 20 and 70 years, and are at substantially increased risk for future breast cancer. Substantially increased risk, for the purpose of this study, is defined as a remaining lifetime risk of at least 20%, or risk in the next 10 years of at least 5%^18^ using any of the three validated risk prediction models commonly used in Australia (ie, iPrevent,^32^ Tyrer Cuzick^33 34^ or the Breast and Ovarian Analysis of Disease Incidence and Carrier Estimation Algorithm (BOADICEA),^35 36^) or a personal history of lobular carcinoma or atypical hyperplasia, or previous thoracic irradiation before age 35 years (and given at least 5 years prior).^37^

Clinicians who see eligible women in the catchment clinics and/or refer to the PCMed Service during the study period will also be recruited to the study to assess the acceptability of the PCMed Service intervention.

#### Exclusion criteria for the study population

Women who have ever taken RRMeds for breast cancer prevention, or who have had a bilateral mastectomy, are ineligible. The PMCC has an established, highly specialised, multidisciplinary Risk Management Clinic^38^ for women who carry (or who are at 50% risk of carrying) a pathogenic variant in a high-risk breast cancer predisposition gene (such as *BRCA1*, *BRCA2*, *PALB2* or *TP53*). Women who are eligible for the PMCC Risk Management Clinic are thus not eligible to attend the PCMed Service (or for this study). Some women with pathogenic variants in moderate-risk genes such as *CHEK2* and *ATM* are eligible for referral to the PCMed Service if they are deemed not eligible for the PMCC Risk Management Clinic.

Clinicians from catchment clinics will be excluded from the study if they are no longer working in the clinic at the end of the study period.

### Outcome measures and data collection

#### Effectiveness of the PCMed Service intervention (primary aim)

The use of RRMeds by similar women before and after the implementation of the PCMed Service will be assessed 12 months after attendance at a catchment clinic. To determine the use of RRMeds prior to the implementation of the PCMed Service, a retrospective chart review is collecting data on consecutive eligible women (historical controls) who attended catchment clinics prior to the commencement of the PCMed Service. For women who attended familial cancer centre catchment clinics, the BOADICEA risk prediction model^35 36^ is routinely applied, and each individual’s risk is documented in their medical record. Other catchment clinics did not consistently apply risk prediction models. Therefore, the first author (KLW) applies one of the three prediction models at the time of data collection (with the choice of prediction model dependent on which is most appropriate based on the available data). First author (KLW) extracts the following data from the medical record for each woman: date of birth, indigenous status, country of birth, socioeconomic status, marital status, parity, education level, whether an interpreter was required, catchment clinic attended, type of clinician seen in the catchment clinic (eg, geneticist, medical oncologist), date of last catchment clinic consultation, whether RRMeds were discussed at that consultation and whether the client had commenced RRMeds within 12 months after the last consultation in the catchment clinic. Comparable data are being collected for the prospective group of women who attend the catchment clinics after commencement of the PCMed Service, regardless of whether they attend the new PCMed Service. For historical controls and those in the prospective cohort, if the first author, KLW, is unable to find evidence of a discussion about RRMeds in the woman’s electronic medical record (clinic notes and correspondence), it is presumed that RRMeds were not commenced. It is noted that not all clinicians might routinely document everything discussed in a consultation. Therefore, the prescription history in the medical record is also reviewed. If a discussion about RRMeds is documented, or the prescription history indicates that a RRMed was prescribed, women are contacted to ascertain if RRMeds were commenced within 12 months of that consultation.

#### Effectiveness of the PCMed Service (secondary aims)

Secondary outcomes include the self-reported use of RRMeds by clients and the reason for not using RRMeds at 6 months after the PCMed Service consultation, where a decision was made regarding whether or not to take RRMeds. These data are obtained through a follow-up telephone call and include (where relevant) whether RRMeds were commenced, the medication commenced, the date of commencement, whether RRMeds were ceased and the reason for ceasing. For women who did not commence RRMeds, data are collected on the reason(s) for not using RRMeds, their intent to use RRMeds in the future, the reason for not intending to use RRMeds (if relevant) and factors that would facilitate use of RRMeds.

#### Adoption of the PCMed Service

To what extent the PCMed Service intervention is adopted will be measured by the attendance of clients at the Service within 6 months following a documented discussion about the Service at the relevant catchment clinic.

#### Acceptability of the Service

Quantitative and qualitative data are collected to explore acceptability for both clients and clinicians. Acceptability of the Service to clients is measured using a brief survey based on the theoretical framework of acceptability (TFA).^39^ This comprises seven constructs (affective attitude, self-efficacy, burden, intervention coherence, perceived effectiveness, ethicality and opportunity cost) that guide the assessment of a client’s perception of acceptability.^39^ The survey is sent to clients 2–3 weeks after the PCMed Service consultation where a decision was made on whether to take RRMeds or not. If no response is received, two reminders are sent out at 1–2 weeks, and then 3–4 weeks after the initial invitation was sent.

A subset of clients are invited to participate in semistructured phone interviews after their last consultation in the PCMed Service. The purpose is to interrogate the acceptability survey findings of the PCMed Service for the study sample. An interview guide was developed based on the constructs of the TFA^39^ thought to be most relevant to the PCMed Service intervention (ie, affective attitude, self-efficacy, burden, intervention coherence and perceived effectiveness). The interview guide was pilot tested on the first two clients, with questions subsequently refined.

A purposive sampling framework, using quota sampling,^40^ will be used to ensure a representative cross-section of the sample population based on age (20–39 years, 40–55 years and >55 years), residence (defined using the Accessibility/Remoteness Index of Australia^41^) and referring clinic. To achieve ‘theoretical saturation’, it is estimated that a sample size of 15–20 clients is needed. However, saturation will be assessed through preliminary analysis, and data collection will continue until saturation is reached.^42 43^

Acceptability of the Service to clinicians will also be measured using a brief survey based on the TFA.^39^ A subset of clinicians will be invited to participate in a semistructured telephone interview. A separate interview guide for clinicians, based on the constructs of the TFA,^39^ has been developed which will further explore clinicians’ experiences and the acceptability of the PCMed Service, including the referral process and any interactions with the Service. The interview guide will be piloted with the first clinician and refined as required. A purposive sampling framework has been devised, using quota sampling^40^ to ensure a representative cross-section of clinicians based on clinic type, specialty and sex (male/female). Clinicians will be recruited until saturation of themes is reached, and it is estimated that a sample size of 15–20 clinicians is needed.^42 43^

#### Feasibility and fidelity of the PCMed Service

Data are being collected on the number of consultations delivered by telehealth versus face-to-face versus telephone; the type of clinician leading the consultation (nurse practitioner versus medical oncologist); the duration of each consultation; how many consultations were required before a prescription was written for RRMeds or the client was discharged from the Service; the number of clients who failed to attend the planned postprescription consultation and the self-reported reason; the number of additional postprescription reviews required. Utilisation of the telephone hotline service is also being recorded, including the number of calls received, the duration of each phone call, the caller type (eg, client, referring clinician and PCP) and the self-reported reason for the call. Any unintended consequences or deviations from the planned processes in the delivery of the PCMed Service are being documented by the nurse practitioner in a research journal as the events occur.

#### Cost of the PCMed Service

The cost to deliver the PCMed Service will be evaluated by comparing the average funding received per client who attended the PCMed Service with the average cost per client of providing the Service.

Services provided in Australian public hospitals, including PMCC, are funded using an activity-based funding model,^44^ with payment based on service activity (ie, the number and type of service provided).^44^ It uses national classifications for services and prices, all set in Australia by the Independent Health and Aged Care Pricing Authority.^45^ The PCMed Service activity is captured in the PMCC database management systems. The Service activity will be extracted at the end of the study period, and a monetary figure allocated to the activity so the funding received can be calculated.

Data used to calculate the cost of service delivery will include the number and duration of all consultations (including calls to the hotline telephone service) and the attending clinician (nurse practitioner or medical oncologist). Cost per client consultation is calculated by multiplying the duration of the consultation by the hourly cost of the nurse practitioner or medical oncologist, respectively. A reasonable estimate of time spent by an administration officer per client (eg, making appointments, sending notifications) will be used. If an interpreter was used, the cost of the interpreter service will be added. Consultation cost, administration officer cost and interpreter cost will be summed into a total cost per patient.

### Statistical considerations and analyses

#### Sample size considerations

The prospective sample size required for the primary outcome will depend on the uptake of RRMeds in the historical cohort. We will identify 100 consecutive historical controls. Based on Australian data,^25^ we expect the uptake of RRMeds in the historical cohort to be 2%. Based on the clinical judgement of the investigators, we consider an increase from a baseline of 2%, prior to the commencement of the PCMed Service, to 20% after its implementation to be clinically significant enough to warrant implementation of this complex intervention. A prospective sample size of 57 clients who attend the PCMed Service from catchment clinics will be required to provide 80% power to detect a minimum change from 2% to 20% at a 5% significance level. To account for a possible 10% loss to follow-up, we will recruit a sample of 63 consenting clients from catchment clinics as they attend the PCMed Service. If the uptake of RRMeds (in the historical cohort) is substantially different from 2%, the prospective sample size will be adjusted.

Recruitment to the Service and the study commenced on 14 November 2023. To date, 100 women (historical controls) have been recruited to the retrospective component of the study and 278 women to the prospective component of the study. Thirty-five of the prospectively recruited women are eligible for the analysis of the primary outcome; the remainder are eligible for other outcome analyses.

#### Descriptive statistics

Categorical variables will be summarised using frequency and percentage. Continuous variables will be summarised using mean, SD and/or SE and range; or median, IQR and range as appropriate. Event data will be presented as point estimates with associated 95% CI presuming an underlying Poisson, negative binomial or zero-inflated distribution as appropriate.

#### Inferential statistics

Comparisons of event proportions between the prospective cohort and historical controls will be conducted using a χ^2^ test, Fisher’s exact test or linear mixed modelling as appropriate. Associations between participant characteristics and study outcomes will be explored using univariable and multivariable logistic, Poisson and/or negative binomial regression and/or linear mixed modelling as indicated. Multivariable models will be assessed for collinearity and interactions between explanatory covariates. Adjustment for clinic effects will be conducted by including the clinic identifier as a random effect in the aforementioned models. The final selection of test or model type will be confirmed on initial review of the collected data, with consideration given to the number of available events for each outcome. For all analyses, p<0.05 will be considered significant. All analyses will be undertaken using R statistical computing software, V.4.4.2^46^ and/or Stata, statistical software, V.18.^47^

#### Analysis sets

The analysis for the primary outcome will include all clients from catchment clinics who attend the PCMed Service and historical controls. A sensitivity analysis will consist of all eligible women attending catchment clinics from the commencement of the study period, regardless of whether they attended the PCMed Service, and historical controls.

#### Analysis of the primary outcome

##### Effectiveness of the PCMed Service intervention

The proportion of women who start RRMeds within 12 months of a catchment clinic consultation will be summarised preimplementation and postimplementation of the PCMed Service using descriptive statistics. Event proportions will be compared using a χ^2^, Fisher’s exact test or linear mixed modelling as indicated. Differences in the distribution of confounders between the historical and prospective cohorts will be managed using univariable and multivariable logistic, Poisson and/or negative binomial regression and/or linear mixed modelling as indicated.

### Analysis of secondary outcomes

#### Effectiveness of the PCMed Service

Descriptive statistics will be used to summarise the proportion of women (without contraindications^24^) who used RRMeds within 6 months of their PCMed Service consultation and the reason for non-use of RRMeds at 6 months after the initial consultation at PCMed Service, where a decision was made whether to take RRMeds.

#### Adoption of the PCMed Service

The proportion of women who attended each catchment clinic and who subsequently attended the PCMed Service within 6 months of a catchment clinic consultation will be summarised using descriptive statistics. Associations between participant characteristics and the likelihood of adoption will be analysed using logistic regression. This analysis will use the sensitivity analysis. Descriptive statistics will be used to summarise the reason(s) for declining a referral to the PCMed Service as an exploratory endpoint.

#### Acceptability of the Service

TFA metrics will be summarised using descriptive statistics. Interview recordings will be transcribed professionally, and transcripts checked against audio recording for accuracy and deidentification prior to analysis. Deidentified transcript data will be managed using NVivo^48^ software. The lead analyst will familiarise themselves with the data by reading each transcript multiple times. Interview data will be analysed using an inductive approach informed by the TFA constructs. It is anticipated that the majority of the inductive codes will align with the TFA constructs, however, we will prioritise capturing women’s experience with the service by starting with inductive coding. We will then map codes to the concepts of the TFA. To ensure academic rigour, two investigators will independently code the first six interviews and determine the level of agreement on coding the data. After interview 15, new interviews will be reviewed in light of the coding framework to determine when saturation of the main themes is achieved. The final coding framework will be reviewed by all investigators.

#### Feasibility and fidelity of the PCMed Service

Evaluation of service delivery will be summarised using descriptive statistics. The research journal will be analysed using the framework for reporting adaptations and modifications-expanded.^49^

#### Cost of the PCMed Service

The cost will be evaluated by determining the difference between the funding received and the cost of service delivery for clients attending the PCMed Service. A sensitivity analysis will consist of calculating the difference between funding received and the cost of service delivery for two client subgroups: clients attending the PCMed Service for consultation but who decline RRMeds, and clients attending the PCMed Service who take up RRMeds. Funding received and the cost of service delivery will be summarised using descriptive statistics.

## Discussion

The findings of this process evaluation will provide valuable insights into the effectiveness of the novel PCMed Service in initiating the appropriate use of RRMeds for women at increased risk of a first breast cancer. If successful, the intervention could ultimately have a substantial impact on increasing RRMeds usage, which has been shown to reduce the incidence of oestrogen receptor-positive breast cancer by up to 60%.

Our study will also explore the factors influencing the adoption and acceptability of the PCMed Service for both clients who attend the Service and clinicians. This is crucial for understanding the barriers to service engagement. Previous research has highlighted the lack of confidence among primary care providers in discussing and prescribing RRMeds, suggesting that a telehealth service with specialist support may be an effective solution. By assessing both qualitative and quantitative data on acceptability, we aim to identify strategies to improve uptake of the PCMed Service and to refine its delivery.

Furthermore, evaluating the feasibility, fidelity and cost of the PCMed Service is vital to ensure its sustainability and to inform future implementation strategies and future economic evaluations. We expect that the PCMed Service, if successful, could be replicated in other regions, offering an accessible and scalable model for breast cancer prevention.

In conclusion, this evaluation of the PCMed Service will contribute to closing an important evidence–implementation gap in breast cancer prevention and could inform future public health strategies for women at increased risk of the disease.

## Patient and public involvement

We are grateful for the contribution of our consumer representative (SMi) and representatives from the local Primary Health Network. SMi assisted in the design of the intervention (PCMed service), development of the research protocol, review of study materials for participants, (including a patient information consent form, the TFA surveys for women and clinicians, correspondence with clients, and client resources) and testing of all processes for clients following receipt of referral to the PCMed Service. The Service and planned concurrent evaluation were presented to the Primary Care and Population Health Committee at PMCC, and we are grateful for the valuable input received from representatives on this committee. A representative from Primary Health Network has reviewed all correspondence developed for PCPs.

## Ethics and dissemination

Ethics approval for this study has been granted by the PMCC Human Research Ethics Committee (EC00235): HREC/101142/PMCC.

Retrospective and prospective data collected on clients attending catchment clinics are obtained under a waiver of consent, as approved by the PMCC Human Research Ethics Committee. This is because it is impractical to consent these women, and there is sufficient protection of their privacy and confidentiality as names will not be used, and reporting of the study results will not allow individuals to be identified. Clients attending the catchment clinics who then attend the PCMed Service have the option to ‘opt out’ of having their data collected and analysed. All clients attending the PCMed Service, regardless of the referring clinic, are invited to participate in the study (including a survey, follow-up telephone calls and an optional interview) via a patient information and consent form (online supplemental file 1). Clinician consent to use data collected about them is implied if the clinicians return the survey and/or participate in the interview.

Manuscripts detailing research findings will be submitted to peer-reviewed journals for publication. Data will be presented at national and international conferences and scientific meetings. In addition, results will be disseminated to stakeholders through relevant specialty networks (by way of newsletters, webinars and educational meetings). For example, breast surgeons will be notified through the Breast Surgeons of Australia and New Zealand Society^50^ by way of an electronic newsletter. Family Cancer Clinicians will be notified through the Kathleen Cuningham Foundation Consortium for Research into Familial Breast Cancer website.^51^ Australian PCPs will be notified through the Royal Australian College of General Practitioners^52^ by way of educational webinars.

## Supplementary material

10.1136/bmjopen-2024-098198 online supplemental file 1
