# Supplementary material for: Evaluation of a telehealth service to support breast cancer prevention medication uptake: a protocol of a mixed methods study
Source: BMJ Open. 2025 Jun 18;15(6):e098198. doi: 10.1136/bmjopen-2024-098198 (PMC12182191; doi:10.1136/bmjopen-2024-098198)
Supplement: online supplemental file 1 [file bmjopen-15-6-s001.pdf]

Participant Information Sheet and Consent Form

**Title**	Supporting uptake of breast cancer prevention medications and minimising treatment discontinuation: a process evaluation
**Project Number**	23/162L
**Project Sponsor**	Peter MacCallum Cancer Centre
**Principal Investigator**	Professor Kelly-Anne Phillips

---

1. Introduction

We are inviting you to take part in the *Supporting uptake of breast cancer prevention medications and minimising treatment discontinuation: a process evaluation* research project because you will be attending to the Preventing Cancer with Medications (PCMed) Service. The PCMed Service is a new Service that provides personalised information to women at increased risk of future breast cancer to help them decide if they want to take daily tablets for 3 to 5 years to reduce their chance of getting breast cancer. These tablets are often referred to as risk-reducing medications.

This research project will help determine how useful the PCMed Service is and whether any changes are needed to improve it. Involvement in this research requires written consent. This **Participant Information Sheet** tells you about the research project and explains what is involved.

We ask you to read this **Participant Information Sheet** carefully and ask questions about anything that you don't understand or that you want to know more about.
When you feel comfortable you can sign the **Consent Form** at the end of this document.

Your participation is voluntary

Participation in this research is completely voluntary. If you don't wish to take part, you don't have to. Importantly, if you do not wish to take part in this research, this does not affect your ability to access the PCMed Service or your future care in any way.

Your withdrawal from the study

If you decide to take part and later change your mind, you are free to withdraw from the project at any stage. Your decision whether to take part or not, or to take part and then withdraw, will not affect your care or your relationship with staff at the PCMed Service or the Peter MacCallum Cancer Centre.

You can withdraw from the project at any time by completing and signing the 'Participant Withdrawal of Consent Form'. This is provided at the end of this document and is to be completed by you and returned to the research team only if you chose to withdraw.

If you decide you want to take part in the research project, you will be asked to sign the consent section. By signing this section, you are telling us that you:

- understand what you have read;
- consent to take part in the research processes that are described;
- consent to the use of your personal and health information as described.

You will be given a copy of this Participant Information Sheet and Consent Form to keep.

2. What is the purpose of this research?

The purpose of this research is to learn if the PCMed Service is effective, deliverable as planned, and acceptable to women and clinicians. While you will not receive any direct benefit from the research, the results will inform future improvements to the Service. The research project may provide important information to support the possible development of similar services across Australia.

Guidelines in Australia and internationally, all recommend that women who are at increased risk of breast cancer consider the use of risk-reducing medications. However, our previous research has shown that many doctors don't think they have enough knowledge to discuss these medications with women. This may be a major reason why the use of these tablet medications is very low in Australia.

This research project will take place at the Peter MacCallum Cancer Centre and is being led by Professor Kelly-Anne Phillips who is an expert in breast cancer prevention.

3. What does participation in this research involve?

For the purposes of this research, we will need to collect information from you. We would like your permission to collect information from:

1. Your PCMed Service appointments
2. A PCMed Service experience questionnaire
3. A PCMed Service experience telephone interview
4. PCMed Service follow-up telephone calls

Your PCMed Service appointments

During your appointment/s, information will be collected including your health history, your personal risk of breast cancer, whether you would consider taking risk-reducing medications now or in the future, and if you would not consider taking these tablets, the reasons why.

PCMed Service experience questionnaire

Shortly after your initial PCMed appointment/s, you will be sent an invitation by email or post to complete a brief questionnaire asking about your experience with the PCMed service. This questionnaire should take you about 5 minutes to complete.

PCMed Service experience telephone interview

After the questionnaire has been received by the research team, you may be contacted to participate in a telephone interview to help us gain a deeper understanding of your experience. This interview will be scheduled at a time that suits you and should take about 20 minutes. This

interview will be audio recorded and then typed-up. The typed-up documents will be de-identified and analysed by the researchers. This telephone interview is entirely optional, and you will have the opportunity to decline.

PCMed Service follow-up phone calls

You will receive a telephone phone call 6 months after your initial appointment/s with the PCMed Service. This telephone call will take approximately 5 to 10 minutes, and we will collect information about your decision, experience and thoughts around risk-reducing medications including whether you intend to take the tablets in the future, and if there are reasons that would make you consider taking the tablets in the future.

You will receive another telephone call at 12 months from the time you attended the appointment at the clinic that referred you to the PCMed Service. This phone call will likely be about 10 to 12 months after you attend the PCMed Service. This telephone call will take about 5 minutes. We will collect information on whether you have taken risk-reducing medications and whether you plan to take these tablets in the future.

You or your doctor may contact our Service at other times during the research project. To help us learn about the delivery of the Service, we will collect information on all the telephone calls received, including who called, the reason and duration of the call, who was able to assist with the call (i.e., administration officer, research assistant, nurse practitioner or medical oncologist). If you require additional appointments, information will also be recorded on the reason an appointment was required, the type of the appointment (for example by telephone or telehealth), who the appointment was with (nurse practitioner or medical oncologist), the duration and reason for the appointment.

There are no costs associated with participating in this research project, nor will you be paid.

Participants will receive a letter at the conclusion of the research project informing them of the findings.

4. What are the possible risks and disadvantages of taking part?

Participating in this research project will require some time as outlined above. Otherwise, there are no substantial risks or disadvantages.

We have been careful to make sure that the questions in the PCMed Service experience questionnaire or that are asked during the PCMed Service experience telephone call are unlikely to cause you any distress. During the telephone interview, if you do not wish to answer a question, you may ask the interviewer to move on or you may stop immediately. If you become upset or distressed as a result of your participation in the research project, the research team will be able to arrange for counselling or other appropriate support. Any counselling or support will be provided at no cost and by qualified staff who are not members of the research team.

5. What will happen to information about me?

By signing the consent form you agree to the research team collecting and using personal and health information about you for this research project.

The research information will be kept strictly confidential according to the National Statement on Ethical Conduct in Human Research (2007) and the Australian Code for Responsible Conduct of Research (2007).

We will store your name and email address in the same secure research database as the other information we are collecting about you. This is necessary so that we can send you email invitations to complete the study questionnaire. When it is time for research data to be exported from the database and analysed, identifying information including your name and email address will not be exported. At the conclusion of this research and as soon as it is possible to do so, your name and email address will be deleted from the database altogether. Data will then be stored in a coded form (re-identifiable) so relevant information can be linked. You will not be identified during analysis or in any publication resulting from this research.

Audio-recordings (from the interview) will be kept on a password-protected computer. These recordings are typed-up and will be analysed in a coded form so no individual taking part in the interviews can be identified. Your information will be stored on a secure central database controlled by the Peter MacCallum Cancer Centre and any paper records will be kept in a locked cabinet with access only available to the researchers who are directly using your data.

Participant and research data will be stored in the research database for a period of at least 5 years from the last publication (as per the Australian Code for Responsible Conduct of Research Guidelines). After 5 years these files will be destroyed by erasure unless further approval for retention is obtained.

It is anticipated that the results of this research project will be published and/or presented in a variety of forums. Individual participants will not be identifiable in any of the presented or published material.

In accordance with relevant Australian and/or Victorian privacy and other relevant laws, you have the right to request access to the information about you that is collected and stored by the research team. You also have the right to request that any information with which you disagree be corrected. Please inform the research team member named at the end of this document if you would like to access your information.

6. Who has reviewed the research project?

All research in Australia involving humans is reviewed by an independent group of people called a Human Research Ethics Committee (HREC). The ethical aspects of this research project have been approved by the HREC of the Peter MacCallum Cancer Centre.

This project will be carried out according to the *National Statement on Ethical Conduct in Human Research (2007)*. This statement has been developed to protect the interests of people who agree to participate in human research studies.

7. Further information and who to contact

The person you may need to contact will depend on the nature of your query. If you want any further information concerning this project or if you have any problems which may be related to your involvement in the project, you can contact the researcher on *03 8559 8264* or any of the following people:

Research contact person

Name	Katrina West
Position	Nurse Practitioner
Telephone	(03) 8559 8632
Email	PCMedService@petermac.org

The details of the Peter MacCallum Cancer Centre complaints person are:

Complaints contact person

Position	Consumer Liaison
Telephone	(03) 8559 7517

If you have any complaints about any aspect of the project, the way it is being conducted or any questions about being a research participant in general, then you may contact:

Reviewing HREC approving this research and HREC Executive Officer details

Reviewing HREC name	Peter MacCallum Cancer Centre Ethics Committee
HREC Executive Officer	Ethics Coordinator
Telephone	(03) 8559 7540
Email	ethics@petermac.org

Consent Form

**Title**	Supporting uptake of breast cancer prevention medications and minimising treatment discontinuation: a process evaluation
**Project Number**	23/162L
**Project Sponsor**	Peter MacCallum Cancer Centre
**Principal Investigator**	Professor Kelly-Anne Phillips

<u>Declaration by Participant</u>

I have read the Participant Information Sheet, or someone has read it to me in a language that I understand.

I understand the purposes, processes and risks of the research described in the project.

I have had an opportunity to ask questions and I am satisfied with the answers I have received.

I freely agree to participate in this research project as described and understand that I am free to withdraw at any time during the project without affecting my future care.

I understand that I will be given a signed copy of this document to keep.

I consent to taking part in the research project.

Name of Participant (please print) ______________________________

Signature ______________________ Date ______________________

<u>Declaration by Researcher†</u>

I have given an explanation of the research project, its procedures, and risks and I believe that the participant has understood that explanation.

Name of Researcher† (please print) ______________________________

Signature ______________________ Date ______________________

† An appropriately qualified member of the research team must provide the explanation of, and information concerning, the research project.

Note: all parties signing the consent section must date their own signature.

Participant Withdrawal of Consent Form

**Title**	Supporting uptake of breast cancer prevention medications and minimising treatment discontinuation: a process evaluation
**Project Number**	23/162L
**Project Sponsor**	Peter MacCallum Cancer Centre
**Principal Investigator**	Professor Kelly-Anne Phillips

<u>Declaration by Participant</u>

I wish to WITHDRAW my participation in the research project effective from the date below. I request the research team handles the information they have collected about me in the following way (choose one option):

- ☐ DESTROY all information collected about me so it can be no longer used in any research. Please note the information collected about you as part of your clinic appointments will be retained in your medical record as it will be required in order to provide you with appropriate clinical care. However, it will be removed from the research database and not be used by the researchers.
- ☐ RETAIN all information collected about me to date so it can continue to be used for this research.

I understand that:

- ☐ no further information about me will be collected for the research project from the withdrawal date;
- ☐ information about me that has already been analysed by the research team may not be able to be destroyed; and
- ☐ choosing to withdraw from the study will not affect my access to the PCMed Service or other Health Services.

ONLY sign this IF you decide to WITHDRAW CONSENT from the Research Project

Name of Participant (please print) ____

Signature ______________________________ Date ______________________________

This form should be forwarded by email to: PCMedService@petermac.org

Alternatively, forms can be posted to:
Professor Kelly Phillips
Peter MacCallum Cancer Centre
Locked Bag 1, A'Beckett St
Melbourne Vic 8006
