# Supplementary material for: Evaluation of a telehealth service to support breast cancer prevention medication uptake: a protocol of a mixed methods study
Source: BMJ Open. 2025 Jun 18;15(6):e098198. doi: 10.1136/bmjopen-2024-098198 (PMC12182191; doi:10.1136/bmjopen-2024-098198)
Supplement: Supplementary file 2 [file bmjopen-2024-098198.reviewer_comments.pdf]

PEER REVIEW HISTORY

BMJ Open publishes all reviews undertaken for accepted manuscripts. Reviewers are asked to complete a checklist review form and are provided with free text boxes to elaborate on their assessment. These free text comments are reproduced below.

ARTICLE DETAILS

Title (Provisional)

EVALUATION OF A TELEHEALTH SERVICE TO SUPPORT BREAST CANCER PREVENTION MEDICATION UPTAKE: A PROTOCOL OF A MIXED METHODS STUDY

Authors

West, Katrina Louise; Spelman, Tim; Cui, Wanda; Latham, Sarah; Deij, Sabine; Minck, Sandy; Mason, Sarah; Saunders , Christobel; Poliness, Cathie; James, Paul A.; Wheeler, Greg; Keogh, Louise A; Best, Stephanie; Phillips, Kelly Anne

---

VERSION 1 - REVIEW

**Reviewer**	**1**
**Name**	**DeCensi, Andrea**
**Affiliation**	**E.O. Ospedali Galliera**
**Date**	**31-Jan-2025**
**COI**	**None**

The low uptake of risk-reducing medications is a critical barrier in attempting to reduce the risk of breast cancer. This study addresses this barrier through an innovative telehealth-based approach, making an important contribution to the field.

The study is well-designed, feasible, and proposed by a distinguished group of researchers.

I look forward to seeing the results of this study, which could serve as a valuable model for other groups aiming to implement similar strategies."

The title accurately reflects the content but could be more concise by removing redundant terms. For example: "Evaluation of a Telehealth Service to Support Breast Cancer Prevention Medication Uptake: A Mixed Methods Study.

**Reviewer**	**2**
**Name**	**Nabi, Hermann**

Affiliation Hôpital du Saint-Sacrement

Date 24-Mar-2025

COI None

This is a well-crafted protocol, but there are a few sections that require further elaboration and clarification.

The authors stated that "Substantially increased risk, for the purpose of this study, is defined as a remaining lifetime risk of at least 20%, or risk in the next 10 years of at least 5% (using a validated risk prediction model), or a personal history of lobular carcinoma (LCIS) or atypical hyperplasia (AH), or previous thoracic irradiation before age 35 years (and given at least 5 years prior)"

Several questions arise here:

1. Which specific risk prediction model will be used, or is there a plan to select one?

2. Will the same model be applied consistently across all clinics involved?

3. What medications are considered as "RRMeds" in this context?

4. Were women informed about the potential adverse effects of these medications?

Additionally, in another section, the authors state: "For historical controls and those in the prospective cohort, if there is no recorded discussion about RRMeds in the electronic medical record, it is presumed that RRMeds were not commenced. If a discussion about RRMeds is documented, women are contacted to confirm if RRMeds were started within 12 months of that consultation." It is unclear how the documentation of a discussion about RRMeds will be verified. This needs further clarification.

The protocol mentions that time-to-event data will be summarized and visualized using Kaplan-Meier estimates, but it is not clear which specific objective this refers to. The section on descriptive and inferential statistics is too general, making it difficult to determine which analyses are linked to the study's objectives. It would be beneficial to be more specific in this section.

Finally, the knowledge transfer (KT) or mobilization activities mentioned appear to be traditional scientific outputs. Do the authors plan any additional or more innovative KT activities? This could be expanded upon for clarity

---

Reviewer 3

Name Lauzier, Sophie

Affiliation Laval University

Date 26-Mar-2025

COI Sophie Lauzier has received unrestricted research grants from Pfizer Canada.

A MIXED METHODS PROCESS EVALUATION OF A CLINICAL SERVICE TO SUPPORT THE UPTAKE OF BREAST CANCER PREVENTION MEDICATIONS BY WOMEN AT INCREASED RISK OF THE DISEASE

Bmjopen-2024-098198

SUMMARY

This is a protocol for an ongoing study aimed at evaluating a telehealth service (PCMed) that provides expertise to women at high risk of breast cancer regarding breast cancer risk-reducing medications (RRMeds). The primary outcome is the comparison between the proportion of women who initiated RRMeds in the year before (historical controls) and the year after the implementation of PCMed. A total of 57 women will need to be included in PCMed and 100 historical controls to detect a statistically significant change in the proportion of women with an RRMeds prescription, from 2% to 20%. Secondary outcomes will be assessed among women who were referred to or used PCMed, specifically focusing on the adoption, acceptability, feasibility, fidelity, and costs of the service.

GENERAL COMMENTS

The study addresses the important issue of breast cancer prevention in high-risk women. The protocol is comprehensive, well-structured, and clearly presented. The methodology is generally robust, particularly as it is grounded in an evaluation framework that encompasses multiple dimensions (e.g., effectiveness, adoption, acceptability) and employs mixed methods. As this is an ongoing funded study, my comments aim to enhance the clarity of the protocol outlined in this manuscript. These comments are listed below.

1) The risk-reducing medications (RRMeds) are a strategy to lower breast cancer risk, but they may also have negative effects, such as side effects. This aspect should be addressed more explicitly in the Introduction.

2) In the Introduction or Discussion, services similar to PCMed, if any, should be mentioned.

3) The study's start date is provided, but the expected date for the end of recruitment should also be specified (or the recruitment status to date).

4) A clear definition of being at substantially increased risk for future breast cancer is provided, but references that support this definition (e.g., clinical guidelines) should be included. Additionally, it is unclear if prediction tools were systematically employed and documented in the medical records during the historical period.

5) Women will be contacted about the initiation and use of their RRMed only if their medical record mentions a discussion about RRMed. Authors should specify whether this

information is routinely documented, particularly for historical controls. If available, pharmacy records could be a more objective and precise source of information.

6) For the qualitative study among clients, it is mentioned that quota sampling will be used. The characteristics that will be considered to perform the sampling should be specified.

7) In the Data Analysis section, Kaplan-Meier estimates are mentioned, but it is unclear which objective this relates to (i.e., which objective will involve time-to-event analysis).

8) The authors have set an increase from 2% to 20% in RRMeds uptake as clinically significant. It should be specified on what basis is this effect size determined (e.g. clinical observations, previous studies).

9) On page 16, the proportion of women without contraindications is mentioned. These contraindications should be presented and mentioned if this information is available for both the retrospective and prospective cohorts.

VERSION 1 - AUTHOR RESPONSE

REVIEWER: 1

Dr. Andrea DeCensi, E.O. Ospedali Galliera

Comments to the Author:

1. The title accurately reflects the content but could be more concise by removing redundant terms. For example: "Evaluation of a Telehealth Service to Support Breast Cancer Prevention Medication Uptake: A Mixed Methods Study.

Response

Please see our response to the editor, question 1.

REVIEWER: 2

Dr. Hermann Nabi, Hôpital du Saint-Sacrement, Université Laval

Comments to the Author:

1. Which specific risk prediction model will be used, or is there a plan to select one?

Response

Any one of three validated risk prediction models will be used. The text (page 10, "marked copy") now reads: "*Substantially increased risk, for the purpose of this study, is defined as a remaining lifetime risk of at least 20%, or risk in the next 10 years of at least 5%*[18] *(using* ***any of the three*** *validated risk prediction model****s commonly used in Australia (i.e., iPrevent[32], Tyrer Cuzick[33,34] or BOADICEA[35,36]****)"*

This information is also provided in "Figure 2 Legend, A:Pre-Service work-up" (page 34, "marked copy").

2. *Will the same model be applied consistently across all clinics involved?*

<u>Response</u>

The application of the risk prediction models has now been outlined by the addition of the following text (page 12, "marked copy"): ***"For women who attended Familial Cancer Centre Catchment Clinics, the BOADICEA risk prediction model[35,36] is routinely applied and each individual's risk documented in their medical record. Other Catchment Clinics did not consistently apply risk prediction models. Therefore, the first author (KW) applies one of the three prediction models at the time of data collection (with choice of prediction model dependent on which is most appropriate based on the available data)".***

3. *What medications are considered as "RRMeds" in this context?*

<u>Response</u>

We consider RRMeds to be any of the medications referred to in the introduction (paragraph 3, page 6, "marked copy"). *"These medications include selective estrogen reception modulators (SERMs), tamoxifen and raloxifene, and aromatase inhibitors (AIs), anastrozole and exemestane……"*

We have revised the text (page 11, "marked copy") and added the words "for breast cancer prevention" to make this more explicit.

"Women who have ever taken RRMeds ***for breast cancer prevention****, or who have had a bilateral mastectomy are ineligible"*.

4. *Were women informed about the potential adverse effects of these medications?*

We acknowledge that it may not be clear that we discuss the potential adverse effects of these medications. Therefore, we have revised the text to make this more explicit. In the introduction (page 7, "marked copy") we have added details of the potential adverse effects of these medications.

"Common adverse effects include gynaecological and vasomotor symptoms, menstrual changes, and less commonly, more serious events such as thromboembolism (with SERMs) or endometrial cancer in post-menopausal women (with tamoxifen)[6], or bone loss (with AIs)[10]."

In addition we have replaced the word "risk" with "any potential adverse effects" in the text (page 34, "marked copy"). *".....The names and mechanism of action of potentially appropriate RRMed(s), the absolute risk reduction that could be achieved with RRMeds, other benefits and* ***any potential adverse effects*** *of RRMeds is discussed".*

5. Additionally, in another section, the authors state: "For historical controls and those in the prospective cohort, if there is no recorded discussion about RRMeds in the electronic medical record, it is presumed that RRMeds were not commenced. If a discussion about RRMeds is documented, women are contacted to confirm if RRMeds were started within 12 months of that consultation." It is unclear how the documentation of a discussion about RRMeds will be verified. This needs further clarification.

Response

Documentation about RRMeds is verified by reviewing the clinic notes and correspondence in the woman's electronic medical record. The text (page 12, "marked copy") has been revised to make this more explicit.

"For historical controls and those in the prospective cohort, ***if first author, KW is unable to find*** *evidence of a discussion about RRMeds in the* ***woman's*** *electronic medical record* ***(clinic notes and correspondence)*** *it is presumed that RRMeds were not commenced.* ***It is noted that not all clinicians might routinely document everything discussed in a consultation. Therefore, the prescription history in the medical record is also reviewed.*** *If a discussion about RRMeds is documented, or the*

***prescription history indicates that a RRMed was prescribed,** women are contacted to ascertain if RRMeds were commenced within 12 months of that consultation."*

6. The protocol mentions that time-to-event data will be summarized and visualized using Kaplan-Meier estimates, but it is not clear which specific objective this refers to.

Response

We thank the reviewer for this observation. We confirm that no outcomes are time-to-event and thus Kaplan-Meier statistics are not indicated in this analysis. We have now removed this reference (page 17, "marked copy") from the manuscript.

7. The section on descriptive and inferential statistics is too general, making it difficult to determine which analyses are linked to the study's objectives. It would be beneficial to be more specific in this section.

Response

We thank the reviewer for this helpful comment and have revised the section **"Statistical Considerations and Analyses"** (page 16, "marked copy") previously titled **"Data Analysis and Sample Size Considerations"** to distinguish statistical considerations from outcome-specific analyses and explicitly align each analysis with the relevant study outcome. The revisions include:

- Moving of content related to sample size calculations and definition of analysis sets from *"*Analysis of the primary outcome" "*Effectiveness of the PCMed Service"* (page 19, "marked copy") (previously named *"*Effectiveness of the PCMed Service – Primary Outcome") into two new sub-sections: *"*Sample size considerations*"* (page 16, "marked copy") and *"*Analysis sets*"* (page 18, "marked copy").
- The content in *"*Analysis of the primary outcome" "*Effectiveness of the PCMed Service Intervention"* has been revised to clearly describe the statistical methods (page 19, "marked copy"). The test now reads: *"The proportion of women who start RRMeds within 12 months of a Catchment Clinic consultation will be summarised pre- and post-implementation of the PCMed Service using descriptive statistics. Event proportions will be* ***compared using a chi-square,***

Fisher's exact test or linear mixed modelling as indicated. *Differences in the distribution of confounders between the historical and prospective cohorts will be managed using* ***univariable and multivariable logistic, Poisson and/or negative binomial regression and/or linear mixed modelling as indicated.***

- A new sub-heading **"Analysis of secondary outcomes"** has been added (page 19, "marked copy").
- A sentence has been added to the "Inferential Statistics" section (commencing page 17, "marked copy") to inform readers that the specific analyses for each aim are provided in the dedicated outcome sections below. The additional text states "The specific analyses to be used to address each of the primary and secondary outcomes is detailed below."

8. ***Finally, the knowledge transfer (KT) or mobilization activities mentioned appear to be traditional scientific outputs. Do the authors plan any additional or more innovative KT activities? This could be expanded upon for clarity.***

Response

The additional innovative KT activities have now been added (paragraph 3, page 23, "marked copy"). The additional text states: "In addition, results will be disseminated to stakeholders through relevant specialty networks (by way of newsletters, webinars and educational meetings). For example, breast surgeons will be notified through the Breast Surgeons of Australia and New Zealand (BreastSurgANZ) Society[50] by way of electronic newsletter. Family Cancer Clinicians will be notified through the Kathleen Cuningham Foundation Consortium (kConFab) for Research into Familial Breast Cancer website[51]. Australian PCPs will be notified through the Royal Australian College of General Practitioners (RACGP)[53] by way of educational webinars."

REVIEWER: 3

Dr. Sophie Lauzier, Laval University

1) ***The risk-reducing medications (RRMeds) are a strategy to lower breast cancer risk, but they may also have negative effects, such as side effects. This aspect should be addressed more explicitly in the Introduction.***

Response

Please see response to Reviewer 2, question 4, at the end of page 3 and on page 4 of this document.

2) ***In the Introduction or Discussion, services similar to PCMed, if any, should be mentioned.***

Response

We have revised the text in (paragraph 1, page 8, "marked copy") to provide clarity around the novelty of the service.

"The PCMed ***Telehealth*** *Service at the Peter MacCallum Cancer Centre (PMCC) in Melbourne,* ***is the first of its kind in Australia****.* ***It is*** *co-led by a medical oncologist (KAP) and nurse practitioner (KW). It* ***is the only*** *specialist* ***service in Australia solely dedicated to*** *deliver****ing*** *comprehensive personalised information to women to facilitate their informed decision-making about using RRMeds."*

3) ***The study's start date is provided, but the expected date for the end of recruitment should also be specified (or the recruitment status to date).***

Response

We have now provided the current recruitment status on (page 17, "marked copy").

*"Recruitment for the study began on November 14, 2023. "****To date, 100 women (historical controls) have been recruited to the retrospective component of the study and 278 women to the prospective component of the study. Thirty-five of the prospectively recruited women are eligible for the analysis of the primary outcome; the remainder are eligible for other outcome analyses."***

4) A clear definition of being at substantially increased risk for future breast cancer is provided, but references that support this definition (e.g., clinical guidelines) should be included.

Response

Reference 18 has been added in the text (page 10, "marked copy").

Additionally, it is unclear if prediction tools were systematically employed and documented in the medical records during the historical period.

Response

Please see response to Reviewer 2, questions 1 and 2, on page 2 and page 3 of this document.

5) Women will be contacted about the initiation and use of their RRMed only if their medical record mentions a discussion about RRMed. Authors should specify whether this information is routinely documented, particularly for historical controls. If available, pharmacy records could be a more objective and precise source of information.

Response

We have added text to acknowledge that all clinicians might not routinely document whether they discussed RRMeds in a consultation and acknowledge that pharmacy records are a more objective and precise source of information: The text (page 12, "marked copy") now reads:

*"............. **It is noted that not all clinicians might routinely document everything discussed in a consultation. Therefore, the prescription history in the medical record is reviewed.** If a discussion about RRMeds is documented, **or the prescription indicates that a RRMed was prescribed,** women are contacted to ascertain if RRMeds were commenced within 12 months of that consultation.*

6) For the qualitative study among clients, it is mentioned that quota sampling will be used. The characteristics that will be considered to perform the sampling should be specified.

Response

We have now added the following words: "based on age (20-39 years, 40-55 years, >55 years), residence (defined using the Accessibility/Remoteness Index of Australia[40]), and referring clinic" to specify the characteristics that will be considered to perform sampling. The text (page 14, "marked copy") now states:

"...........*to ensure a representative cross section of the sample population* ***based on age (20-39 years, 40-55 years, >55 years), residence (defined using the Accessibility/Remoteness Index of Australia[40]), and referring clinic."***.

7) In the Data Analysis section, Kaplan-Meier estimates are mentioned, but it is unclear which objective this relates to (i.e., which objective will involve time-to-event analysis).

<u>Response</u>

Please see response to Reviewer 2, questions 6, on page 5 of this document.

8) The authors have set an increase from 2% to 20% in RRMeds uptake as clinically significant. It should be specified on what basis is this effect size determined (e.g. clinical observations, previous studies).

The effect size was determined by the clinical judgment of the investigators. We have revised text to reflect this (page 16, "marked copy"):

"Based on the clinical judgment of the investigators*, we consider an increase from a baseline of 2%, prior to the commencement of the PCMed Service, to 20% after its implementation to be clinically significant****, enough to warrant implementation of this complex intervention.****"*

9) On page 16, the proportion of women without contraindications is mentioned. These contraindications should be presented and mentioned if this information is available for both the retrospective and prospective cohorts.

<u>Response</u>

We have now added reference 24 for contraindications (page 19, "marked copy").

"*Descriptive statistics will be used to summarise the proportion of women (without contraindications[24])*. In addition, we have outline contraindications in the "INTRODUCTION" (page 6, paragraph 3, "marked copy"). Text now reads:

"Guidelines recommend the consideration of RRMeds in individuals who are at moderate to high risk of developing a future breast cancer[18-23] ***,excluding those with contraindications such as pregnancy, planning pregnancy in the next 3 years, breastfeeding, prior bilateral mastectomy, or use of hormone therapy. Additional***

contraindications include prior thromboembolic events, endometrial cancer (for tamoxifen), smoking, taking anticoagulants or a strong CYP2D inhibitor (for SERMs) and premenopausal status or osteoporosis (for AIs)[24].”

ADDITIONAL MINOR EDITS MADE

The affiliation for author SMi has been corrected.

Minor adjustments to text and addition of references including, reference 24 (page 29, “marked copy”), reference 31 (page 31, “marked copy”) and references 50 – 53 (page 32, “marked copy”) to accommodate changes in response to reviewers.

VERSION 2 - REVIEW

**Reviewer**	**2**
**Name**	**Nabi, Hermann**
**Affiliation**	**Hôpital du Saint-Sacrement**
**Date**	**27-May-2025**
**COI**	

The authors have thoroughly addressed all of my comments. Thank you!
